# Augmenting the Referral Pathway for Retinal Services Among Patients With Diabetes Mellitus at Reiyukai Eiko Masunaga Eye Hospital, Nepal: Protocol for a Nonrandomized, Pre–Post Intervention Study

**DOI:** 10.2196/33116

**Published:** 2021-12-17

**Authors:** Ruchi Shrestha, Prerana Singh, Parami Dhakhwa, Shailaja Tetali, Tripura Batchu, Pragati Shrestha Thapa

**Affiliations:** 1 Department of Vitreoretina Reiyukai Eiko Masunaga Eye Hospital Banepa Nepal; 2 SEVA Nepal Kathmandu Nepal; 3 Department of Research Indian Institute of Public Health Hyderabad India

**Keywords:** diabetes mellitus, diabetic retinopathy, Nepal, health education, study protocol

## Abstract

**Background:**

Diabetic retinopathy (DR) is an important public health issue in Nepal with a huge social and economic impact. Despite the availability of retinal services, people may not access them because of the lack of knowledge about DR and poor referral systems. Published studies on referral pathways in Nepal are scarce. Improving DR awareness among general physicians has the potential to address these challenges.

**Objective:**

The aim of this study is to evaluate the effect of a health education intervention on health personnel, establish a referral pathway, and assess the impact of the intervention on the attendance of patients with diabetes mellitus for retinal screening at Reiyukai Eiko Masunaga Eye Hospital in Nepal.

**Methods:**

This is a nonrandomized, pre- and postintervention study. Health education on DR will be provided to selected health personnel of the intervention hospital (Scheer Memorial) using information education and communication (IEC) materials in the form of PowerPoint presentations, posters, pamphlets, videos, and pre- and postevaluation questionnaires along with referral slip. Pre- and postevaluation will be undertaken during the study period. Data will be analyzed using MS Excel and Epi Info 7.

**Results:**

The ethical approval for this study has been obtained from the Ethical Review Board of the Nepal Health Research Council (ERB Protocol Registration Number # 582/2020P). The study is expected to be completed in 18 months from the start of the project. The baseline data collection was from June to January 2020 for a period of 8 months. The postintervention data collection was from February to September 2021 for a period of 8 months. The last 2 months are planned for data analysis and report writing.

**Conclusions:**

Health education intervention could be a low-cost solution to improve the awareness, access, and utilization of retinal health care services; this is an understudied topic in Nepal. Working closely with the stakeholders, this study will evaluate the role of health education interventions (which are already validated in other low-income settings) to strengthen referral and reduce the burden of DR in Nepal.

**Trial Registration:**

ClinicalTrials.gov NCT04829084; https://clinicaltrials.gov/ct2/show/NCT04829084

**International Registered Report Identifier (IRRID):**

DERR1-10.2196/33116

## Introduction

### Background

Diabetic retinopathy (DR) is a complication of diabetes mellitus (DM) damaging the retinal vessels. If left untreated, it can lead to blindness [1]. It is observed that more than 75% of people with DM for 20 years or more will have some form of DR and 10% will have retinopathy requiring treatment. Timely screening, early detection, and treatment can reduce the risk of blindness by more than 90% [2].

The worldwide prevalence of DR is 34.6% [3]. Globally, it is the fifth leading cause of visual impairment and the fourth leading cause of blindness. DR is responsible for 4.8% of the 37 million cases of blindness worldwide [4].

DR is an emerging cause of blindness in developing countries such as Nepal. Mishra et al [5] reported that 10% of people with DM had some form of DR in Nepal. The prevalence of nonproliferative DR, proliferative DR, and complete vision loss was 9.1%, 0.5%, and 0.3%, respectively, in Nepal [5]. Awareness about retinopathy remains very poor in Nepal and therefore, there is a need to sensitize the public about diabetic eye diseases [6]. Timely referral of patients with DM to retina care centers for screening is likely to improve early diagnosis of DR [7].

Our organization, Reiyukai Eiko Masunaga Eye Hospital (REMEH), is a nonprofitable community-based hospital in Banepa, Nepal, that provides eye care services to a population of 411,057 in the Kavrepalanchok District. In 2019, the hospital launched the retinal clinic and started retinal care services. Because the uptake of DR screening did not increase as expected, we conducted a problem tree analysis and identified that a poor referral system is one of the major reasons for the low uptake of DR screening at our hospital. Similarly, Piyasena et al [8] identified that the lack of knowledge and awareness about DR, and zero awareness of the importance of regular DR screening and follow-up, combined with poor information on referral pathways, were key elements to improve the uptake of DR in Sri Lanka [8].

Published studies on referral pathways in Nepal are scarce. We aim to see if providing health education intervention to selected health personnel and establishing a referral pathway increase the attendance of patients with DM for retinal screening at REMEH. This is a pilot study as no such study was previously conducted in Nepal.

### Research Objective

The aim of this study is to increase retinal screening uptake among patients with DM and to decrease DR-associated blindness, by augmenting the referral pathway in a selected hospital in Nepal.

### Hypothesis

Providing DR health education to selected health personnel and creating a referral pathway will increase the uptake of retinal services (screening and treatment) by patients with DM referred to REMEH.

## Methods

### Study Design, Setting, and Participants

This is a nonrandomized, pre- and postintervention study without a control group. The study was performed in REMEH. This study included selected health personnel of Scheer Memorial Hospital (the intervention hospital) who are directly involved in providing health services to patients with DM. A total of 19 health personnel of Scheer Memorial Hospital fit the inclusion criteria (4 physicians, 4 pediatricians, 8 medical officers, and 3 assistants).

### Sampling Techniques

This involved complete enumerations of all health personnel managing patients with DM in the intervention hospital.

### Inclusion and Exclusion Criteria

All health personnel involved in the management of patients with DM at the intervention hospital were included. Health personnel in the intervention hospital who are not directly involved in managing patients with DM were excluded.

### Intervention Hospital

Scheer Memorial Hospital is the intervention hospital. The retinal physician and the optometrist/outreach coordinator of REMEH will conduct the intervention. The assistant manager of the hospital will be responsible for the logistics. There is no control group in this study.

### Outcome

#### Primary Outcome

This includes change in the proportion of referred patients from Scheer Memorial to REMEH compared with baseline referrals before the intervention.

#### Secondary Outcome

This includes change in the knowledge level of the health care personnel who participated in health education sessions in Scheer Memorial.

### Materials

The following information education and communication (IEC) materials developed by the Indian Institute of Public Health, Hyderabad, India, will be used for intervention after converting its content into Nepalese: PowerPoint slides, poster (Multimedia Appendix 1), pamphlet (Multimedia Appendices 2 and 3), and video. A referral slip (Multimedia Appendix 4) will also be used.

### Pilot Study

We will conduct a pilot study 1 week before the intervention with a group of doctors managing patients with DM in another hospital. The pilot study will help to see if the materials are well understood and the duration of sessions are optimal. Based on the feedback, we will make necessary changes to the IEC material and training delivery method before the intervention.

### Implementation of Intervention

Ethical approval has been obtained from the Nepal Health Research Council. Written informed consent has been obtained from all participants. All selected health personnel of Scheer will visit REMEH for the health education sessions, which will be implemented once a month for 3 months, lasting an hour during the weekends. The session will begin with a preassessment test to know participants’ baseline knowledge about DR. We will deliver a PowerPoint presentation explaining the burden of DM globally and in Nepal. It will include the effect of uncontrolled DM on eyes and possible damage to the retina, risk of DR, and the preventive measures for the early detection and management of DR to avoid vision loss. We will explain to the participants the contents of educational pamphlets. Lastly, we will present the educational video on DR. At the end of the first session, we will hand over the posters, pamphlets, and referral slips to the participants. We will request all 19 participants to distribute pamphlets to patients, counsel them to have fundus examination, and to use referral slips while referring patients to REMEH. We will provide posters related to DR with a request to display them in the waiting area of Scheer Memorial, and at the end of the intervention, we will conduct a quiz and postintervention assessment of the participants. The intervention plan is outlined in Table 1.

Actions expected from selected health personnel in Scheer Memorial:

- Counsel patients with DM to visit REMEH.
- Use the referral slip while referring patients to REMEH.
- Handover DR-related pamphlets to patients with DM.
- Record total cases referred to REMEH in a month.

**Table 1:** The intervention plan with IEC^a^ materials used during each visit.

Visit	Materials	Questionnaire(s)
Pilot	All IEC materials	Pre- and postassessment questionnaires
First	PowerPoint presentations, posters, referral slips	Preassessment questionnaire
Second	Video presentation, pamphlets	—
Third	Refresher of the previous session and a small quiz	Postassessment questionnaire

^a^IEC: information education and communication.

### Data Collection

Data will be collected before and after the health education at Scheer Memorial; besides, patient referral data at REMEH will be collected. All data will be entered into MS Excel every week. The data variables and tools are mentioned in Multimedia Appendices 1-5.

### Data Analysis

The proportional increase of patients referred as a result of the intervention compared with baseline data will be calculated. Change in knowledge of health personnel will be assessed with pre- and postassessment questionnaires prepared using Public Health Foundation of India’s “Certificate Course in Evidence Based Management of Diabetic Retinopathy assessment questionnaire” as a guide (Multimedia Appendix 5). The first 5 questions will be on DM and its complications and the remaining 5 questions will be specific to DR. The Diabetic Retinopathy Awareness Index will be calculated based on the scoring made by Datti et al [9].

Information on DR and duration of DM will be collected, visual acuity will be measured, and stage of DR will be noted to understand the clinical outcomes. Descriptive analysis for the same will be presented. Information on referred data from the intervention hospital will be extrapolated. Mean and SD will be calculated for the demographic variables. The *Z* test will be applied to determine the number of referrals. Paired *t* test will be used for the change in average knowledge score of health care professionals and a chi-square test will be used for pre–post referrals based on the education and experience of health care professions. Data will be analyzed using Epi Info 7.

### Ethical Approval

The ethical approval for this study has been obtained from the Ethical Review Board of Nepal Health Research Council (ERB Protocol Registration Number # 582/2020P).

## Results

The study is expected to be completed in 18 months from the start of the project. The baseline data collection was from June to January 2020 for a period of 8 months. The postintervention data collection was from February to September 2021 for a period of 8 months. The last 2 months are planned for data analysis and report writing. Descriptive analysis will be presented on the demographic variables of the health care professionals, demographic variables of patients, and clinical findings by computing means with SD. The proportion of increase in referral will be calculated by the *Z* test. The change in average knowledge score will be calculated by means and SD. *P* value will be calculated by a paired *t* test. The pre- and postintervention referrals will be determined based on the experience and education of the health care professionals by a chi-square test.

## Discussion

Our study is a nonrandomized, pre- and postintervention design that focuses on health professionals as stakeholders to increase the referral of DR. The stakeholders will be provided health education to create awareness and knowledge about DR using PowerPoint slides, posters, pamphlets, and referral slips. After the intervention, we hope to see increased referral of patients with DR from the intervention hospital (Scheer Memorial) and better knowledge among health care personnel. Different studies have shown that providing health education sessions on DR to physicians and assessing their awareness improved the uptake of screening for DR. Use of a referral slip for communication between an ophthalmologist and physicians was an effective strategy to change the behavior of referring physicians [8-17].

Health education intervention could be a low-cost solution to improve the awareness, access, utilization of retinal health care services, but this is an understudied topic in Nepal. Working closely with the stakeholders (health care professionals), this study will evaluate the role of health education interventions (which are already validated in other low-income settings) to reduce the burden of DR in Nepal.

The strength of our study is that it will be one of the first in Nepal that includes health care professionals involved in DM management and strengthens the referral for DR screening.

Because of COVID-19 restrictions, the number of selected health personnel participating in health education could reduce. We also do not have a control hospital to compare our intervention and the effect of the referral pathway between 2 hospitals, which could limit our study’s generalizability. We need further studies to understand barriers faced by health personnel on the referral process and reasons for delays, if any.

## Appendix

Multimedia Appendix 1 Poster for Diabetic Retinopathy.

Multimedia Appendix 2 Pamphlet for Diabetic Retinopathy.

Multimedia Appendix 3 Pamphlet for Diabetic Retinopathy.

Multimedia Appendix 4 Referral Slip.

Multimedia Appendix 5 Pre- and postquestionnaire.

## Abbreviations

DM: diabetes mellitus
DR: diabetic retinopathy
IEC: information education and communication
REMEH: Reiyukai Eiko Masunaga Eye Hospital
