# Supplementary figures and images for: Augmenting the Referral Pathway for Retinal Services Among Patients With Diabetes Mellitus at Reiyukai Eiko Masunaga Eye Hospital, Nepal: Protocol for a Nonrandomized, Pre–Post Intervention Study
Source: JMIR Res Protoc. 2021 Dec 17;10(12):e33116. doi: 10.2196/33116 (PMC8726041; doi:10.2196/33116)

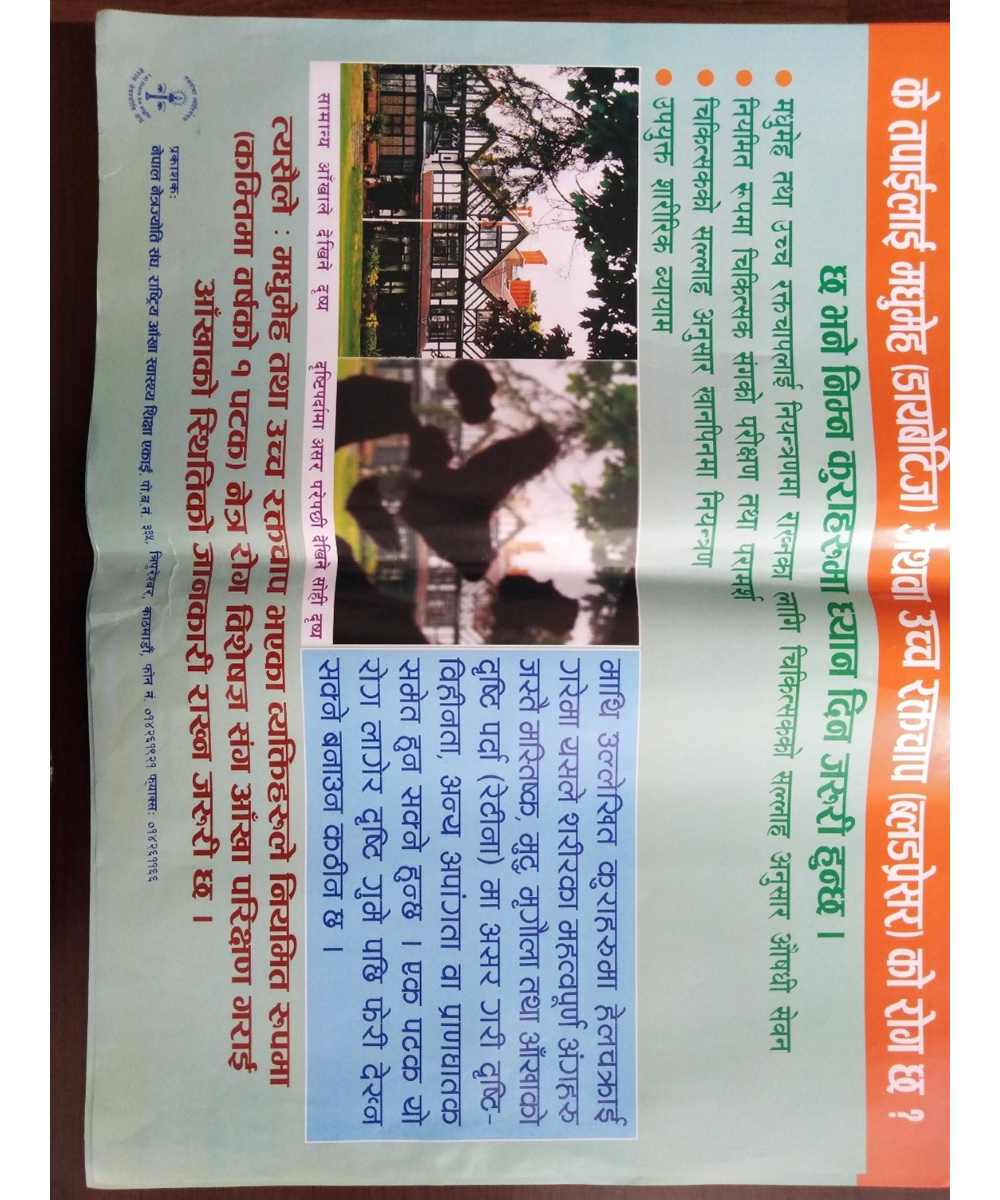

Supplement: Multimedia Appendix 1 [file resprot_v10i12e33116_app1.png]

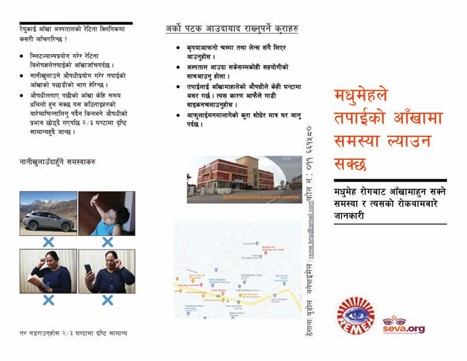

Supplement: Multimedia Appendix 2 [file resprot_v10i12e33116_app2.png]

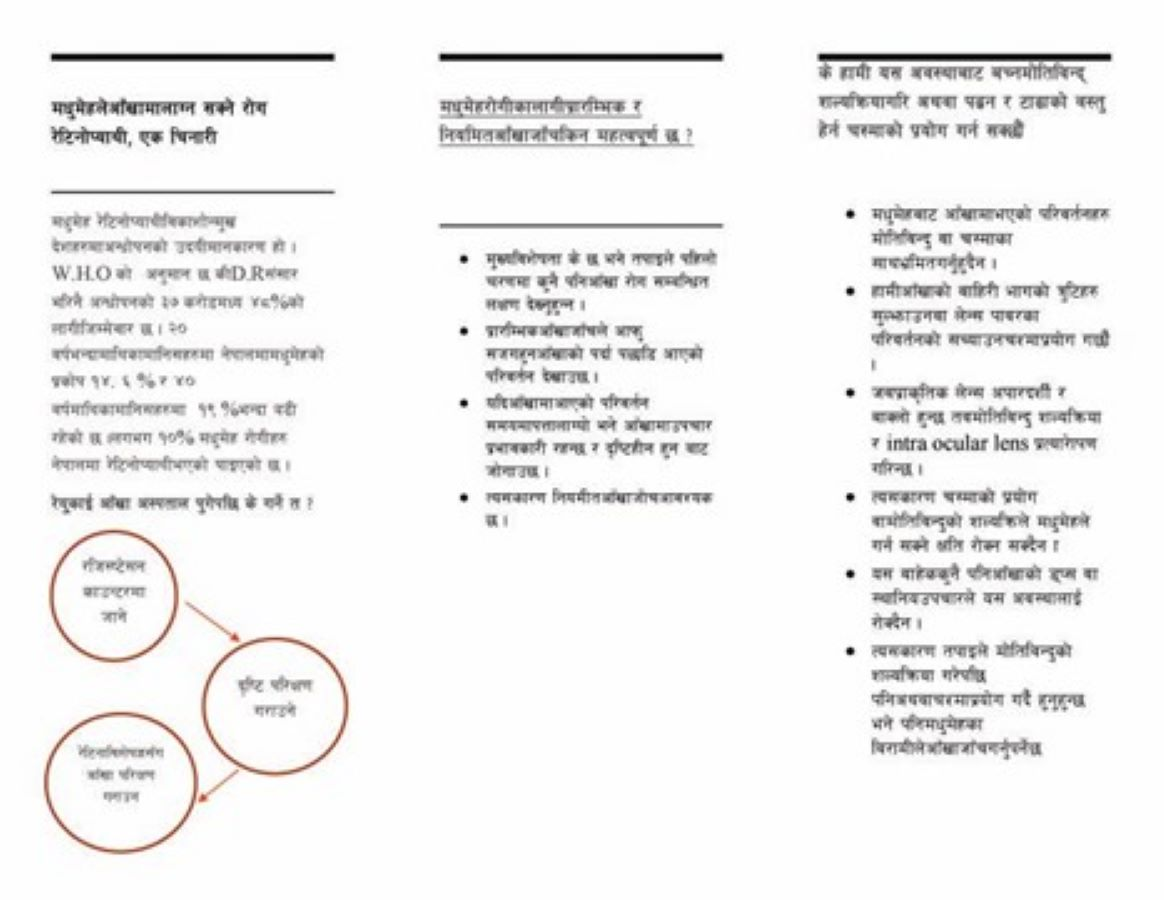

Supplement: Multimedia Appendix 3 [file resprot_v10i12e33116_app3.png]

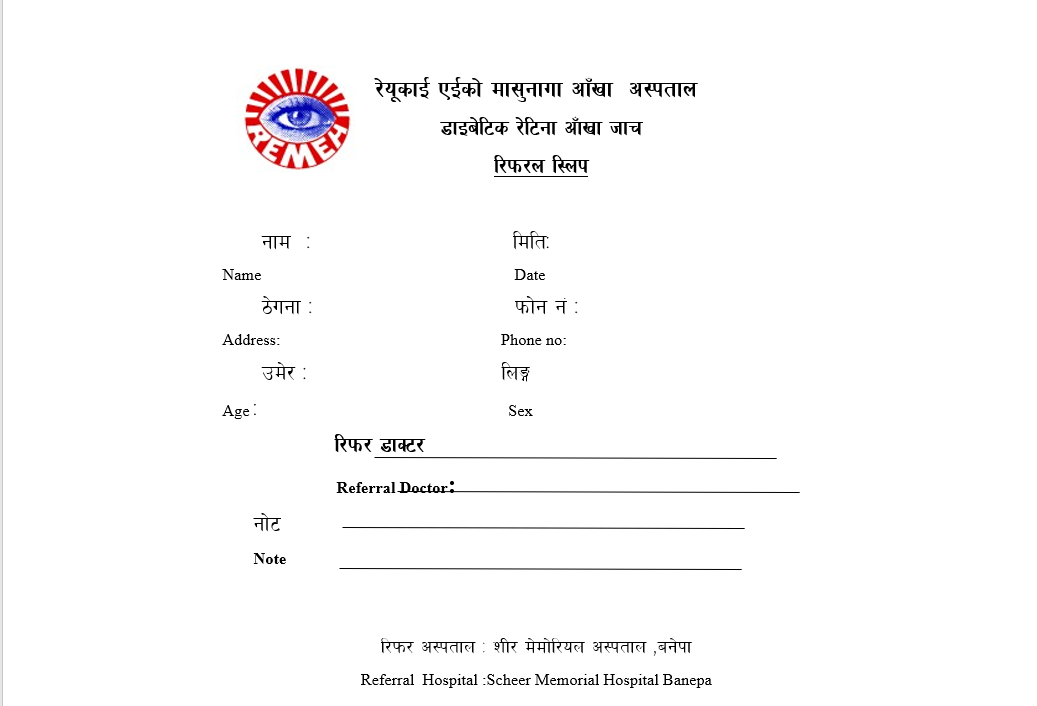

Supplement: Multimedia Appendix 4 [file resprot_v10i12e33116_app4.png]
