# Supplementary material for: Augmenting the Referral Pathway for Retinal Services Among Patients With Diabetes Mellitus at Reiyukai Eiko Masunaga Eye Hospital, Nepal: Protocol for a Nonrandomized, Pre–Post Intervention Study
Source: JMIR Res Protoc. 2021 Dec 17;10(12):e33116. doi: 10.2196/33116 (PMC8726041; doi:10.2196/33116)
Supplement: Multimedia Appendix 5 [file resprot_v10i12e33116_app5.docx]

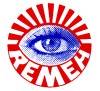


Baseline Questionnaire

Diabetes Eye Care Education Initiative

**PLEASE TICK / CIRCLE THE ANSWERS**

1. **Out of all the Outpatients/Inpatients seen by you, what percentage of people with diabetes you see in a week?**
2. 2% - 5%
3. 5% - 10%
4. 10% - 20%
5. More than 20%
6. **Out of total number of people with diabetes, what percentage constitutes people with Diabetic Retinopathy?**
7. < 5%
8. 5% - 10%
9. 10% - 20%
10. More than 20%
11. **One of the most common causes of blindness in the working population.**
    1. Cataract
    2. Untreated DR
    3. Refractive Error
    4. None
12. **Retinal examination must be done in all people with diabetes, irrespective of the type of diabetes**
13. Once in 3 years
14. Once in 5 years
15. Once a year
16. When the patient has vision complaints
17. **The symptoms produced in diabetic retinopathy are**
18. Watering
19. Itching
20. Redness and discharge
21. Blurred vision
22. **The pathogenesis of diabetic retinopathy is due to**
23. Microvascular occlusion
24. Microvascular leakage
25. Both of the above
26. None of the above
27. **The most important non-modifiable risk factor associated with DR is**
28. Anemia
29. Cardiovascular disease
30. Duration of diabetes
31. Smoking
32. **All patients with diabetic retinopathy require**
33. Laser photocoagulation
34. Glycemic control
35. Intravitreal anti-VEGFs (vascular endothelial growth factor)
36. Ocular surgery
37. **Which of the following is the first sign of diabetic retinopathy?**

a. Hard-exudates

b. Cotton wool spot

1. Micro aneurysm
2. Flame Shaped haemorrhages
3. **Diabetic macular edema is treated with**
   1. Laser
   2. Anti-VEGF
   3. Both of the above
   4. None of the above.
4. **Which of following is an indication for panretinal laser photocoagulation**
   1. Moderate non-proliferative diabetic retinopathy
   2. Proliferative diabetic retinopathy
   3. Mild non-proliferative diabetic retinopathy
   4. Diabetic macular edema
5. **Which of the following is NOT an indication for vitrectomy**
   1. Non-clearing vitreous haemorrhage
   2. Tractional retinal detachment
   3. Proliferative diabetic retinopathy
   4. Diabetic macular edema

***Thank You for your Participation in the survey!***


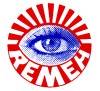


Post Training Questionnaire

Diabetes Eye Care Education Initiative

**PLEASE TICK / CIRCLE THE ANSWERS**

1. **One of the most common causes of blindness in the working population.**
   1. Cataract
   2. Untreated DR
   3. Refractive Error
2. **Retinal examination must be done in all people with diabetes, irrespective of the type of diabetes**
3. Once in 3 years
4. Once in 5 years
5. Once a year
6. When the patient has vision complaints
7. **The symptoms produced in diabetic retinopathy are**
8. Watering
9. Itching
10. Redness and discharge
11. Diminution of vision
12. **The pathogenesis of diabetic retinopathy is due to**
13. Microvascular occlusion
14. Microvascular leakage
15. Both of the above
16. None of the above
17. **The most important non-modifiable risk factor associated with DR is**
18. Anemia
19. Cardiovascular disease
20. Duration of diabetes
21. Smoking
22. **Which of the following is the first sign of diabetic retinopathy?**

a. Hard-exudates

b. Cotton wool spot

1. Micro aneurysm
2. Flame Shaped haemorrhages
3. **All patients with diabetic retinopathy require**
4. Laser photocoagulation
5. Glycemic control
6. Intravitreal anti-VEGFs (vascular endothelial growth factor)
7. Ocular surgery
8. **Diabetic macular edema is treated with**
   1. Laser
   2. Anti-VEGF
   3. Both of the above
   4. None of the above.
9. **Which of following is an indication for panretinal laser photocoagulation**
   1. Moderate non-proliferative diabetic retinopathy
   2. Proliferative diabetic retinopathy
   3. Mild non-proliferative diabetic retinopathy
   4. Diabetic macular edema
10. **Which of the following is NOT an indication for vitrectomy**
    1. Non-clearing vitreous haemorrhage
    2. Tractional retinal detachment
    3. Advanced Proliferative diabetic retinopathy
    4. Diabetic macular edema

***Thank You for your Participation in the survey!***
